# Cystatin C: not just a marker of kidney function

**DOI:** 10.1590/2175-8239-JBN-2019-0240

**Published:** 2020-04-03

**Authors:** Sanduni Fernando, Kevan R. Polkinghorne

**Affiliations:** 1 Monash Health, Monash Medical Center, Department of Nephrology, Clayton, Melbourne, Australia.; 2 Monash University, Department of Medicine, Clayton, Melbourne, Australia.; 3 Monash University, School of Public Health and Preventive Medicine, Department of Epidemiology and Preventive Medicine, Prahran, Melbourne, Australia.

Cystatin C, first described in 1961 and formally named in 1984, is a non-glycosylated, basic protein encoded by the CST3 gene that is found in virtually all nucleated cells. It is a potent inhibitor of lysosomal proteinases and extracellular inhibitors of cysteine proteases that play a pleiotropic role in human vascular pathophysiology, in particular regulating cathepsins, which are overexpressed in atherosclerotic and aneurysmal lesions^1^.

The role of serum cystatin C as a measure of kidney function (filtration) is now well established. Cystatin C is generated at a mostly constant rate, is freely filtered, reabsorbed and catabolized in the proximal tubule of the nephron^2^. It is not affected by body habitus and muscle mass and as such, it is thought to be a more accurate measure of kidney function compared to serum creatinine 3. Standard reference materials are now available for cystatin C such that laboratory assays are standardized with glomerular filtration rate estimating equations (eGFR) developed and validated for routine clinical use^3^. Limitations to its routine use in clinical practice has been primarily cost considerations where, for example, it costs up to ten times that of a serum creatinine assay 4 and it can be affected by thyroid disease, adiposity, and underlying inflammation^1^^,^ 5.

Beyond its use in the estimation of kidney function, cystatin C-based eGFR has been shown to be a superior predictor of cardiovascular disease (CVD) and mortality when compared to serum creatinine-based eGFR^6^. In fact, when combined with albuminuria, cystatin C eGFR added predictive discrimination to routinely used current CVD risk scores whereas serum creatinine-based eGFR did not add additional discrimination^4^. This suggests that cystatin C-based eGFR should be used not only to assess kidney function, but also should be part of an individual’s cardiovascular risk assessment. 

Other potential roles for cystatin C include being an earlier marker for acute kidney injury, a superior marker of kidney transplant function, CVD risk and transplant failure, and a more accurate measure of function in specific sub-populations such as liver cirrhosis and oncology^6^. In this issue of the Brazilian Journal of Nephrology, Moreira *et al*. assess another specific sub-population where cystatin C may have additional utility over serum creatinine and other markers, namely subjects with end stage kidney disease^7^.

Moreira *et al*. assessed serum and peritoneal dialysis effluent cystatin C in 52 patients treated with peritoneal dialysis (PD) in a single dialysis center. In addition to the assessment of residual renal function (RRF) they explored the correlation of cystatin C and cardiovascular events as well as an assessment of body composition using bioimpedance. Like the general population, higher levels of cystatin C was associated with a two-fold increase in the likelihood of cardiovascular disease (odds ratio (OR) 2.65), although the 95% confidence interval was wide, reflecting significant uncertainty in the result (0.84 to 8.37). This is not surprising given the limitations of the small sample size and the cross-sectional nature of the study design and analysis. The assessment of RRF in patients treated with PD is critical given its importance in prognosis and total overall dialysis clearance. The current study reaffirms our understanding that cystatin C may be a good marker of assessing RRF. Patients who have been on PD longer have a higher cystatin C level correlating to the gradual loss of RRF seen after dialysis commencement. Weekly total creatinine clearance was significantly negatively correlated with cystatin C levels as would be expected. It is important to note that PD also removes cystatin C and hence its levels will be determined by both RRF and dialysis clearance. Also as expected, cystatin C levels positively correlated with relative lean tissue mass. 

While the study by Moreira *et al*. raises more questions than it answers due to the size and study design, given the role of cystatin C as an established tool for the assessment of kidney function in the general and CKD population as well as an additive marker of cardiovascular risk assessment, further large prospective studies in the PD population are warranted in order to delineate whether cystatin C will have a role in the management of the PD population. 
